# Trends in Marijuana Use among Adolescents in the United States

**DOI:** 10.3390/pediatric16040074

**Published:** 2024-10-15

**Authors:** Jack Yang, Maria C. Mejia, Lea Sacca, Charles H. Hennekens, Panagiota Kitsantas

**Affiliations:** Department of Population Health and Social Medicine, Charles E. Schmidt College of Medicine, Florida Atlantic University, Boca Raton, FL 33431, USA; jyang2023@health.fau.edu (J.Y.); mejiam@health.fau.edu (M.C.M.); lsacca@health.fau.edu (L.S.); profchhmd@prodigy.net (C.H.H.)

**Keywords:** adolescents, marijuana, cannabis, students, United States

## Abstract

Background: Marijuana is a widely used substance in the United States (US) and worldwide. We explored trends in self-reported marijuana use among US adolescents overall as well as by gender, race/ethnicity, and school grade. Methods: Biennial data from the Youth Risk Behavior Survey from 2011 to 2021 included 88,183 adolescents in grades 9th through 12th. We used percentage change as a measure of effect and the chi-square test for significance. All analyses were conducted at the national level. Results: The percentage of adolescents who reported current marijuana use dropped significantly from 23.1% in 2011 to 15.8% in 2021 (*p* < 0.05). The self-report of trying marijuana for the first time before age 13 also decreased significantly from 8.1% in 2011 to 4.9% in 2021 (*p* < 0.05). For current use, there were similar significant decreases by race/ethnicity, with Asian, Hispanic, and White adolescents experiencing the steepest declines. In 2021, the percentage of Black adolescents self-reporting marijuana use was significantly higher (20.5%) compared to White (14.8%), Hispanic (16.7%), and Asian (5.1%) adolescents. Although current marijuana use declined significantly for both girls and boys over time, in 2021 girls were more likely (17.8%) to currently use marijuana than boys (13.6%). In 2011, the opposite was true, with boys (25.9%) being more likely to use marijuana than girls (20.1%). Conclusions: In US adolescents in 2021, there were decreases in self-reports of marijuana use compared to 2011. Behavioral interventions within school and family environments may be critical in mitigating the risk of marijuana use.

## 1. Introduction

Marijuana has become one of the most frequently used illicit substances among adolescents in the United States (US) [1,2]. Regular or high marijuana usage during adolescence, which constitutes a crucial period for neural development, has been linked to poor cognitive outcomes, such as disadvantaged learning, working memory tests, and attention, even when considering years of school and verbal intelligence [3,4,5,6]. Other detriments associated with marijuana use in adolescents include being 2–3.5 times more likely to report a lower grade point average and a 4-fold increase in adult psychosis diagnoses [7,8]. Research shows that marijuana use negatively impacts brain functioning by decreasing synaptic pruning, which leads to a greater volume of gray matter and overall lowers the efficiency of communication between higher-order areas of the brain [9].

Monitoring trends in marijuana use for adolescents in the US is especially important given the increasing number of US states that are legalizing recreational marijuana and the low perceived risk in this population [2,10]. It is important to examine marijuana use at a national level and by gender and other demographics to gain a better understanding of these trends [11].

In this original research, we utilized the Youth Risk Behavior Survey (YRBS) data from 2011 to 2021 to explore temporal trends in marijuana use among US adolescents overall as well as by gender, race/ethnicity, and school grade.

## 2. Materials and Methods

The YRBS, which is a cross-sectional survey, is provided by the US Centers for Disease Control and Prevention and includes self-reports of marijuana use among adolescents in grades 9 through 12 from 2011 to 2021. The YRBS is conducted biennially among students in grades 9 to 12 who are sampled from randomly selected public and private schools [12]. All students in sampled schools and classes are eligible to participate in these surveys. The YRBS monitors health behaviors in large samples of US adolescents that contribute to their leading avoidable causes of premature morbidity and mortality. This survey includes public, parochial, and private schools across the US. In 2021, the student response rate was 79.1%. Detailed descriptions have been reported previously [12].

The total study sample comprised 88,183 US high school students and included data from 2011 (*n* = 15,031), 2013 (*n* = 13,314), 2015 (*n* = 15,250), 2017 (*n* = 14,386), 2019 (*n* = 13,305), and 2021 (*n* = 16,897).

The exposure variables for self-reported marijuana use, also referred to as pot or weed, included (1) ever used, defined as one or more times during their life; (2) first used before age 13 years; and (3) current use, defined as one or more times during the 30 days before the survey. The survey also included self-reported data on race/ethnicity (Black, Asian, Hispanic/Latino, and White), school grade (9th, 10th, 11th, and 12th), and gender (boy/girl) [13].

Descriptive statistics were conducted using percentages as the measures of effect and used the chi-square test for statistical significance. We considered a two-sided *p* value <0.05 to be statistically significant. All of the data analyses were conducted using the Youth Online system which allows access to the YRBS data for the years 2011–2021 [13].

## 3. Results

**Figure 1:**
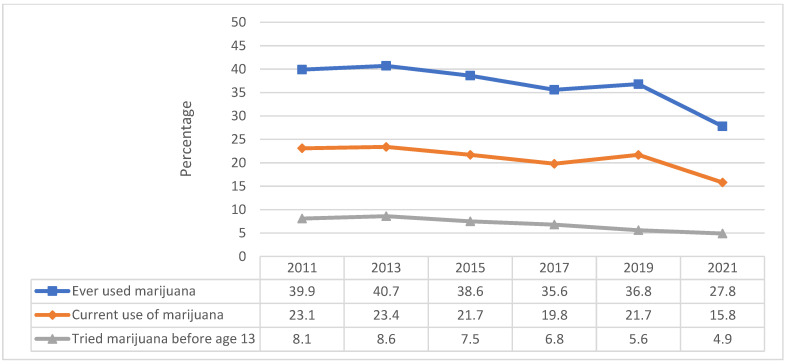
Marijuana use in adolescents, Youth Risk Behavior Survey 2011–2021.

The percentage of adolescents with ever using marijuana significantly decreased from 39.9% in 2011 to 27.8% in 2021 (*p* < 0.05). Similarly, the proportion of adolescents who reported current marijuana use dropped from 23.1% in 2011 to 15.8% in 2021 (*p* < 0.05) (Figure 1). A significant decrease was also observed between 2011 and 2021 for trying marijuana for the first time before age 13 (from 8.1% in 2011 to 4.9% in 2021, *p* < 0.05). Overall, from 2011 to 2013, we observed an increase in use for all these behaviors, a decline from 2015 to 2017 with a slight peak in 2019 (except for tried marijuana before age 13 which continued to decline), and a rapid decrease in 2021.

**Figure 2:**
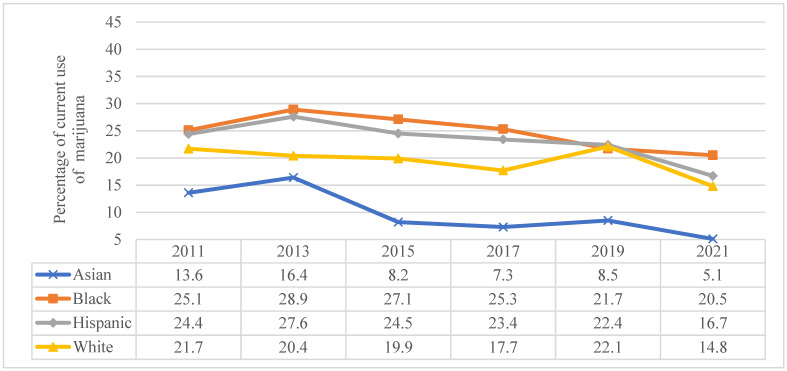
Current marijuana use in adolescents by race/ethnicity, Youth Risk Behavior Survey 2011–2021.

Concerning race/ethnicity, a decline in marijuana use was observed across all racial/ethnic groups between 2011 and 2021 (Figure 2). In 2021, however, the proportion of Black adolescents reporting marijuana use was higher (20.5%) when compared to White (14.8%), Hispanic (16.7%), or Asian (5.1%) adolescents. This was also true for all of the other years except for 2019.

**Figure 3:**
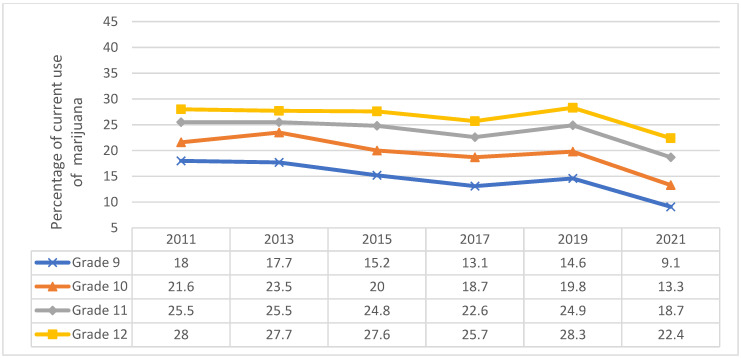
Current marijuana use in adolescents by grade, Youth Risk Behavior Survey 2011–2021.

By grade, in 2021, the current use of marijuana was higher for adolescents in grades 12 (22.4%) followed by those in grade 11 (18.7%) than lower grades (Figure 3). A significant decrease was observed from 2011 to 2021 across all grades, particularly for 9th grade. Overall, a significant decline in current marijuana use was observed across time for all grades except for an increase in 2013 for grade 10 and a slight increase again in 2019.

**Figure 4:**
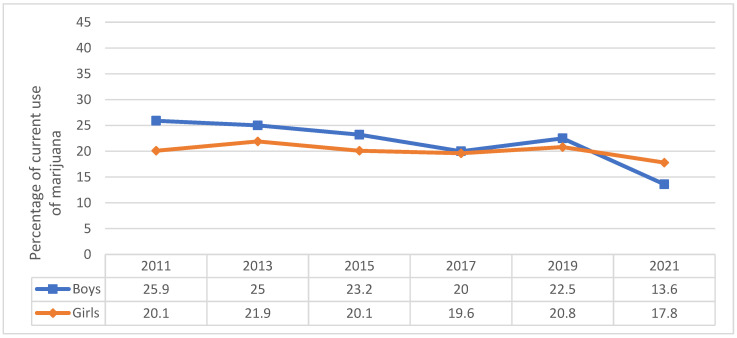
Marijuana use in adolescents by gender, Youth Risk Behavior Survey 2011–2021.

With regard to gender, although current marijuana use declined significantly for both girls and boys over time, in 2021 girls were more likely (17.8%) to currently use marijuana than boys (13.6%) (Figure 4 and Table 1), while the opposite was true for the year 2011, with boys being more likely to use marijuana than girls (25.9% versus 20.1%) (Figure 4). Table 1 displays differences in marijuana use behaviors between boys and girls for 2021, which is the most recent year available. Girls were significantly more likely to report ever using marijuana (30.9%) than boys (24.8%), but no significant differences were observed in marijuana use before age 13. For both girls and boys, Black adolescents followed by Hispanic adolescents were more likely to currently use marijuana, start using marijuana before the age of 13, and have ever used marijuana compared to other racial/ethnic groups. Current use and ever use of marijuana were particularly high among adolescents in grades 11 and 12, and the percentages of use were significantly higher among girls than boys across all grades except for grade 12 (*p*-value < 0.05). Although girls in grades 9 and 11 were significantly more likely to have used marijuana before age 13 (*p*-value < 0.05), boys in grade 12 were more likely (6.4%) than girls (3.2%) to report this behavior (*p*-value < 0.05).

## 4. Discussion

The results of this study show declining trends in marijuana use among US high school adolescents from 2011 to 2021. The decline in marijuana use within this population is part of a broader trend in decreasing substance use during the same period [2,14]. The significant decreases observed in both the “ever used marijuana” and “currently use marijuana” categories highlight a promising reduction in adolescent marijuana use, with usage dropping to approximately 70% of the levels recorded in 2011. Similarly, the percentage of adolescents who tried marijuana before age 13 decreased to about 60% of the 2011 levels. However, despite these encouraging trends, the study reveals persistent disparities in marijuana use when considering demographic factors such as grade level, gender, and race/ethnicity.

While we found a net decline in the percentage of marijuana use among students between 2011 and 2021 for all grade levels, there was consistently higher usage for older grades throughout all years, especially among 12th graders. The consistent observation that 12th graders have the highest rates of marijuana use across all survey years suggests that older adolescents may have greater access to marijuana, possibly due to more developed peer networks and increased independence [15]. As adolescents progress through high school, the likelihood of exposure to marijuana through social circles may increase, contributing to the elevated usage rates among 12th graders. This trend underscores the importance of targeted interventions for older adolescents, who may be at greater risk of regular marijuana use.

One of the most significant findings of this study is the shift in marijuana use trends by gender, with girls surpassing boys in reported marijuana use by 2021. This change is consistent with earlier reports suggesting a narrowing of the gender gap in substance use [16]. The increase in marijuana use among girls could be attributed to evolving social dynamics, including more integrated friend groups where girls may experience greater exposure to marijuana offers from male peers [17]. This trend is concerning given that early initiation and regular use of marijuana during adolescence are associated with an increased risk of developing cannabis use disorder later in life. Future research should explore the underlying factors driving this gender shift and examine the potential long-term implications for female adolescents.

The data also highlight significant racial/ethnic disparities in marijuana use among adolescents. Black adolescents consistently reported higher rates of current marijuana use compared to their Hispanic, White, and Asian peers throughout the study period. This finding aligns with previous research indicating that minority groups, particularly Black adolescents, may perceive lower risks associated with marijuana use, which could contribute to higher usage rates [18]. In contrast, Asian adolescents reported the lowest rates of marijuana use, which may be related to stronger disapproval from parents and peers and higher perceived risks associated with drug use [19]. Addressing these disparities requires culturally sensitive prevention strategies that account for the unique social and cultural factors influencing marijuana use among different racial/ethnic groups.

While the overall decrease in US high school adolescent’s marijuana usage from 2011 to 2021 is encouraging, it is crucial to sustain and build on these gains through continued public health efforts. Behavioral interventions that promote positive connections within school and family environments are essential in mitigating the risk of marijuana use [20]. Interventions such as parenteral communication, monitoring, and modeling, as well as schools providing effective health education and promoting a positive school climate should be further encouraged to continue the decline in marijuana use [21]. Schools should prioritize effective health education programs that clarify the medical benefits and potential harms of marijuana use, particularly as legalization efforts continue to expand across the US [22]. Early prevention programs that focus on delaying the initiation of marijuana use and reducing its appeal to adolescents could further contribute to the downward trend observed in this study.

The findings regarding the increase in marijuana use among girls warrant particular attention. Given the potential for increased vulnerability to the adverse effects of marijuana use, including the risk of developing cannabis use disorder [16], public health initiatives should include targeted messaging and interventions for female adolescents. Understanding the social and environmental factors that contribute to this trend is critical for developing effective prevention strategies.

Despite the strengths of this study, several limitations should be noted. The reliance on self-reported data introduces potential biases, such as social desirability and recall biases, which may affect the accuracy of reported marijuana use. The cross-sectional design limits the ability to establish causality between observed trends and influencing factors. Additionally, the study lacks detailed contextual information on marijuana use, such as frequency and potency, and does not account for variations in state legalization, which may influence trends. While the YRBSS provides a nationally representative sample, the findings may not be fully generalizable to all adolescents, particularly those outside traditional school settings. Moreover, the study does not consider other potential factors, such as socioeconomic status or peer influence, that could impact marijuana use. These limitations highlight the need for further research, especially longitudinal studies, to better understand the factors driving adolescent marijuana use and to inform effective prevention strategies.

## 5. Conclusions

In the US, the current landscape of marijuana legalization in adults adds a complex layer to the issues of adolescent marijuana use. As more states continue to legalize recreational marijuana, the accessibility and perceived normalcy of the drug may increase, particularly for adolescents who may view its legal status as an indication of safety or acceptability. Marijuana legalization can influence adolescent behavior through reduced risk perception and increased availability, which may counteract efforts to reduce use in this vulnerable population.

In conclusion, the data from the YRBS underscore the importance of ongoing surveillance and intervention efforts to reduce marijuana use among US adolescents. By addressing the specific needs of different demographic groups, including grade levels, genders, and racial/ethnic communities, public health initiatives can more effectively combat the risks associated with adolescent marijuana use and promote healthier outcomes for future generations.

## Figures and Tables

**Table 1:** Marijuana use among United States adolescents by gender, Youth Risk Behavior Survey 2021.

	Year 2021		
	Girls % (95% CI)	Boys % (95% CI)	*p*-Value
Current use of marijuana	17.8 (15.8–20.1)	13.6 (12.1–15.3)	0.00
Black	24.8 (20.5–29.6)	16.4 (13.2–20.1)	0.00
Asian	4.7 (2.6–8.3)	5.5 (3.6–8.3)	0.57
Hispanic/Latino	19.1 (15.6–23.1)	13.4 (11.2–15.9)	0.00
White	16.3 (14.4–18.5)	13.6 (11.8–15.5)	0.01
Grade			
9	11.0 (8.8–13.8)	7.2 (5.6–9.1)	0.00
10	16.7 (13.8–20.0)	9.7 (8.1–11.6)	0.00
11	20.8 (17.8–24.1)	16.7 (14.4–19.4)	0.01
12	22.8 (20.0–25.9)	22.0 (19.5–24.8)	0.60
Ever used marijuana	30.9 (28.0–33.8)	24.8 (22.6–27.1)	0.00
Race/Ethnicity			
Black	37.1 (31.1–43.6)	29.7 (24.4–35.7)	0.01
Asian	9.0 (6.5–12.4)	10.5 (7.6–14.2)	0.39
Hispanic/Latino	34.8 (30.7–39.2)	27.4 (22.8–32.4)	0.00
White	29.0 (26.9–31.2)	23.5 (21.3–25.8)	0.00
Grade			
9	17.8 (14.8–21.2)	13.5 (10.8–16.7)	0.01
10	28.1 (24.7–31.8)	20.0 (17.3–23.0)	0.00
11	35.6 (31.3–40.1)	30.7 (26.6–35.1)	0.04
12	42.2 (37.2–47.3)	36.5 (33.3–39.8)	0.06
Marijuana use before age 13	4.7 (3.9–5.8)	4.8 (3.9–5.9)	0.95
Race/Ethnicity			
Black	5.3 (3.6–7.8)	9.2 (7.4–11.4)	0.00
Asian	0.7 (0.4–1.3)	1.6 (0.7–3.4)	0.21
Hispanic/Latino	6.1 (4.0–9.1)	5.6 (4.2–7.4)	0.66
White	4.0 (3.2–5.1)	3.5 (2.6–4.6)	0.39
Grade			
9	5.8 (4.5–7.5)	4.1 (3.0–5.5)	0.01
10	5.0 (3.8–6.6)	3.3 (2.6–4.2)	0.02
11	4.8 (3.9–5.9)	5.2 (3.7–7.2)	0.68
12	3.2 (2.1–4.8)	6.4 (4.9–8.4)	0.00

## Data Availability

The data presented in the study are openly available in Youth Risk Behavior Survey at https://nccd.cdc.gov/Youthonline/App/Default.aspx (15 July 2024).
